# Oral exposure to environmental pollutant benzo[a]pyrene impacts the intestinal epithelium and induces gut microbial shifts in murine model

**DOI:** 10.1038/srep31027

**Published:** 2016-08-09

**Authors:** Céline Ribière, Pierre Peyret, Nicolas Parisot, Claude Darcha, Pierre J. Déchelotte, Nicolas Barnich, Eric Peyretaillade, Delphine Boucher

**Affiliations:** 1 Clermont Université, Université d’Auvergne, EA 4678 CIDAM, F-63001 Clermont-Ferrand, France; 2 Service d’anatomo-pathologie, CHU Clermont-Ferrand, Clermont-Ferrand, France; 3 Clermont Université, Université d’Auvergne, EA 7281 R2D2, F-63001 Clermont-Ferrand, France; 4 Clermont Université, UMR 1071 M2ISH, Inserm/Université d’Auvergne, F-63001 Clermont-Ferrand, France; 5 INRA, Unité Sous Contrat 2018, F-63000, Clermont-Ferrand, France

## Abstract

Gut microbiota dysbiosis are associated with a wide range of human diseases, including inflammatory bowel diseases. The physiopathology of these diseases has multifactorial aetiology in which environmental factors, particularly pollution could play a crucial role. Among the different pollutants listed, Polycyclic Aromatic Hydrocarbons (PAHs) are subject to increased monitoring due to their wide distribution and high toxicity on Humans. Here, we used 16S rRNA gene sequencing to investigate the impact of benzo[a]pyrene (BaP, most toxic PAH) oral exposure on the faecal and intestinal mucosa-associated bacteria in C57BL/6 mice. Intestinal inflammation was also evaluated by histological observations. BaP oral exposure significantly altered the composition and the abundance of the gut microbiota and led to moderate inflammation in ileal and colonic mucosa. More severe lesions were observed in ileal segment. Shifts in gut microbiota associated with moderate inflammatory signs in intestinal mucosa would suggest the establishment of a pro-inflammatory intestinal environment following BaP oral exposure. Therefore, under conditions of genetic susceptibility and in association with other environmental factors, exposure to this pollutant could trigger and/or accelerate the development of inflammatory pathologies.

The gut microbiota is referred as all the diverse microbial populations that inhabit the gastrointestinal tract. Composed of more than 10^14^ bacterial cells displayed over 1,000 species^1^, the intestinal microbial community is considered as a near organ that has coevolved with the human host to perform symbiotic relationship leading to gut homeostasis. Notably, the gut microbiota plays crucial roles in not only energetic balance regulation, drug and xenobiotic metabolism and nutrition but also maturation and modulation of the immune system^2^. Furthermore, the gut microbiota maintains a close relationship with the intestinal mucosa and participates to the regulation of the intestinal barrier effect in a protector and structural role^3^. Although the gut microbiota appears to be largely beneficial to the human host organism, modifications of the bacterial community composition (BCC), or dysbiosis, are associated with digestive system dysfunction that leads to inflammatory bowel diseases (IBD) and other non-digestive chronic disorders, including allergies, arthritic diseases, obesity, metabolic syndromes or autoimmune disorders^4^.

Perturbations of the gut microbiota can be caused by several external factors such as the quality of the environment. The impact of toxic environmental chemical compounds such as cadmium^5^, or polychlorinated biphenyls (PCB)^6^ has been reported in *in vivo* models. Epidemiologic studies have also revealed a relationship between air pollution and human diseases, such as an increased incidence and hospitalization for IBD in industrialized countries^7^,^8^, suggesting a multifactorial origin for these pathologies. In association (or not) with physiological, genetic, immunological and other environmental factors (e.g., stress, diet and antibiotic treatment), the quality of the environment could play a key role in the initiation, development or clinical expression of diseases *via* alterations of the gut microbiota.

Among the different pollutants listed, polycyclic aromatic hydrocarbons (PAHs) are one of the most monitored because most of them have harmful effects in humans. Benzo[a]pyrene (BaP) is the most characterized and toxic member of this pollutant family. Indeed, several mutagenic, carcinogenic and pro-inflammatory effects have been demonstrated in various animal models^9^,^10^. Tumour induction in several organs, including the gastrointestinal tract, led also the BaP to be classified as a group 1 carcinogen in humans by the International Agency for Research on Cancer (IARC)^11^. Human contamination to BaP is unavoidable and occurs through polluted water, soil exposure, as well as through consumption of food, primarily charcoal-broiled, grilled and smoked meats and fishes or poorly cleaned vegetables^12^. The inhalation of polluted air^13^ or tobacco smoke^12^ is another source of BaP exposure. The gastrointestinal tract is consequently the human system with the highest exposure, owing to oral uptakes as well as inhaled BaP, which is transported from the lungs to the digestive system by the mucociliary clearance mechanism^14^.

As it was previously underlined, modifications in gut microbiota have been linked to human diseases^4^, and the toxic effects of BaP have been extensively characterized^9^,^10^. However, the impact of BaP on the intestinal BCC is still unknown. Based on its pro-inflammatory and mutagenic proprieties, we hypothesized that oral exposure to BaP may disrupt the intestinal microbiota, leading to the induction of an abnormal inflammatory response and an alteration of gut functions. Therefore, in the present work, we performed a subchronic oral exposure to BaP in a murine model. 16S rRNA gene high-throughput sequencing analysis of faecal and mucosa-associated microbiota was carried out to investigate bacterial structure shifts (richness, diversity and composition) following BaP oral administration. Furthermore, intestinal inflammation was evaluated via histological observations performed on the intestinal mucosa of both ileum and colon.

## Results

### Development of moderate intestinal inflammation during BaP treatment

**Figure 1:**
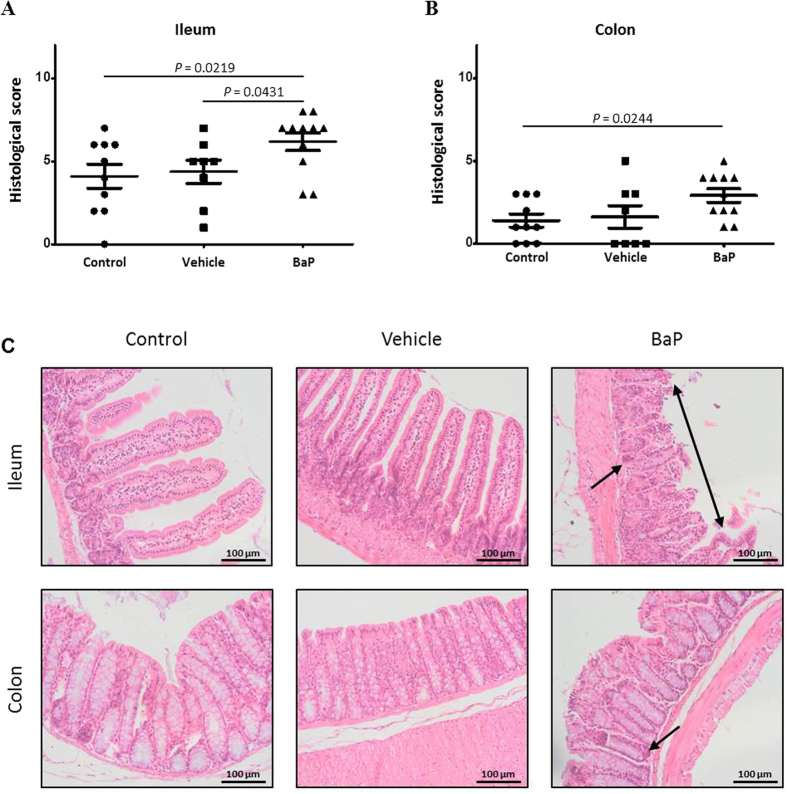
Development of moderate inflammation in mice treated with benzo[a]pyrene (BaP). (**A**,**B**) Histologic scoring of the inflammation and (**C**) representative hematoxylin and eosin histology of ileum and colon of mice orally exposed to sterile physiological saline (control), sunflower seed oil (vehicle) or BaP. Each symbol represents an individual mouse. The values are represented with mean ± SEM and the p-values (**A**,**B**) were derived from a Mann-Whitney test. Epithelial erosion (doubled arrow) and increase of the cellularity in crypts (arrow) are also shown (**C**).

The development of intestinal inflammation in mice was determined by histological examination. Despite that no effect was observed on mouse growth phenotypes (data not shown), moderate inflammatory signs were observed in the ileal and colonic mucosa of C57BL/6 mice exposed to BaP (Fig. 1A,B) compared to the control and vehicle groups, Nevertheless, histological examinations of the mouse intestinal epithelium revealed a significantly lower inflammatory score in the colon than in the ileum (mean histological scores of 2.9 and 6.2 for the colonic and ileal mucosa, respectively) (*p*-value = 0.0013). Infiltration of the epithelium by polynuclear cells was significantly increased in these two intestinal mucosa tissues from mice exposed to orally administered BaP (Fig. S1c,d). Crypt damage and proliferation of inflammatory cells were observed in both ileal and colonic mucosa; in the most severe case, a multifocal erosion of the epithelial surface was also detected (Fig. 1C).

### BaP oral exposure did not alter alpha-diversity indices

We investigated the effects of BaP oral exposure on bacterial richness (qualitative measures [number of operational taxonomic units (OTUs), Chao1]) and diversity (quantitative species-based measures (Shannon) and divergence-based measures (PD))^15^ for different groups of mice. The number of OTUs observed in faecal samples ranged from 3,830 (BaP group - T1) to 4,403 (Control group - T7) (Table S1). However, these values were distant from those estimated by the Chao1 index (ranging from 13,946 ± 953.9 for BaP group, T1 to 16,139 ± 826 for BaP group, T21), indicating that the sequencing depth remained insufficient to evaluate the overall diversity in the present study. BaP oral exposure did not significantly alter the bacterial richness and diversity of the faecal microbiota (Table S1).

Irrespective of the intestinal segment studied, the mucosa-associated microbiota had a significant lower richness than the faecal microbiota, as measured by the mean number of observed OTUs (1,028 ± 206 and 4,138 ± 309 for mucosal and faecal microbiota respectively, *p*-value < 0.0001) (Table S1). As observed for the faecal microbiota, the overall of the bacterial populations was not detected, since the numbers of OTUs were far from those estimated by the Chao1 index (ranging from 3,534 ± 940.9 for BaP group, ileum to 5,054 ± 958.1 for BaP group, proximal colon). Moreover, the alpha-diversity values of the 3 mouse groups did not significantly differ (Table S1).

### Faecal and mucosa-associated microbiota composition

The faecal BCC and its temporal succession for different groups of mice (control, vehicle and BaP) were investigated using 16S rRNA gene sequencing. Regardless of the faecal sample considered, members belonging to Bacteroidetes, followed by Firmicutes and Verrucomicrobia, dominated the BCC at the phylum level (Fig. S2). During this study, 77 bacterial genera were observed among the 55 bacterial families detected, and between 51 and 62 bacterial genera were tracked in each sample (Fig. S2 and Table S2). 40 and 60 bacterial families and genera, respectively, were shared between all of the groups and sampling dates (Fig. S3a). In contrast, 8 bacterial genera were exclusively present in the control group whereas 2 bacterial genera were specifically observed in the BaP treatment group.

The impact of BaP oral exposure on the mucosa-associated microbiota from the ileum, proximal and distal colon was also evaluated. Among all of the mucosal samples combined, 47 bacterial families and 62 bacterial genera were detected (Table S3). In contrast to faecal BCC, Firmicutes followed by Bacteroides (except in the proximal colon of the vehicle group) and Verrucomicrobia predominated the mucosa-associated bacteria (Fig. S5). Whereas 42, 41 and 46 bacterial genera were shared between all experimental groups at the ileum, proximal colon, and distal colon, respectively, some genera were exclusively present in the BaP treatment group in the ileum (*Bacillus*), proximal colon (*Acinetobacter*) and distal colon (unclassified genus of *Erysipelotrichaceae* and *Comamonadaceae*) (Fig. S3b–d).

The bacterial OTUs were classified as abundant (representing greater than or equal to 1% of all of the sequences in each sample) or rare (representing 0.1% or less of all of the sequences in each sample)^16^. Bacterial taxa with relative abundances between 0.1 and 1% were defined as common. Between 13 and 16 bacterial genera dominated the faecal BCC of the 3 mouse groups for each sampling date and represented ≈90% of the relative abundance of the total bacterial populations (Fig. S2). An average of 24 bacterial taxa was defined as rare for each sampling date (Table S2).

### Faecal microbial community shifts during subchronic exposure to BaP

**Figure 2:**
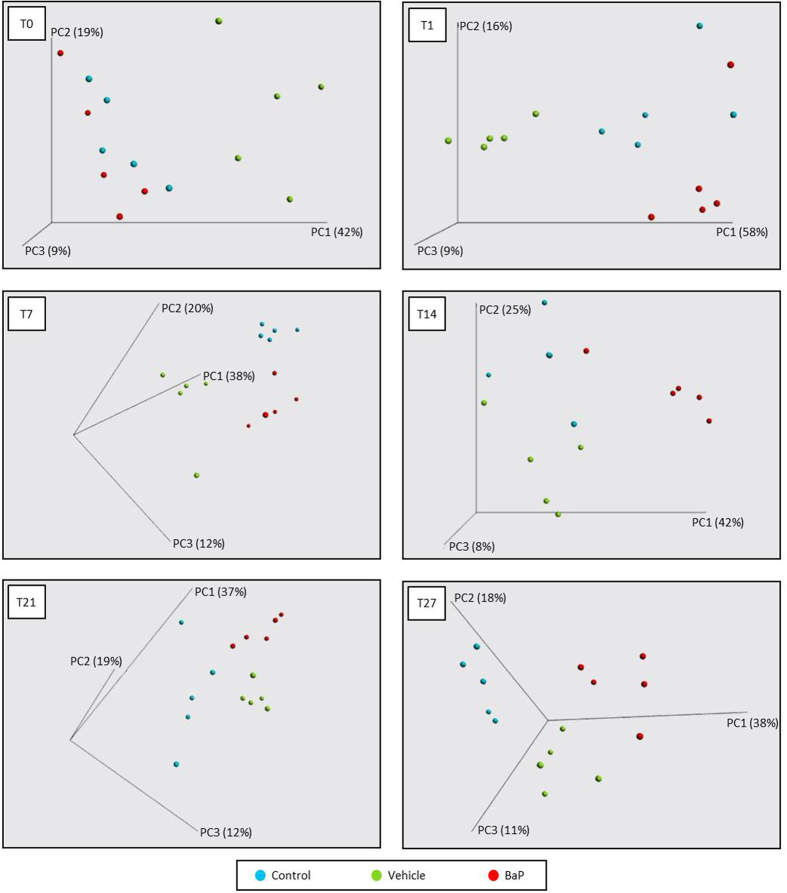
Faecal bacterial patterns of control, vehicle and BaP-treated mice differentiated by principal coordinate analysis (PCoA) on a weighted UniFrac distance matrix. Control, vehicle and BaP-treated mice are represented in blue, green and red, respectively, at different stool collection days: T0 (before exposure), T1 (one day after the first administration), T7, T14, T21 and T27. The R-value from ANOSIM were 0.803 (*p*-value = 0.002), 0.432 (*p*-value = 0.001), 0.684 (*p*-value = 0.001), 0.333 (*p*-value = 0.003), and 0.590 (*p*-value = 0.001) respectively for T1, T7, T14, T21 and T27.

Differences in bacterial taxa composition and abundance were analyzed by using PCoA plots based on a weighted UniFrac distance matrix to compare the BCC of the different mouse groups at each stool collection time (Fig. 2). At T0, surprisingly, the vehicle group mice clustered separately from the control and the BaP groups, indicating that before the beginning of the experiment, the composition and the relative abundance of bacterial communities were already different (Fig. S2). PCoA plot and ANOSIM analysis together showed a clear and significant separation between mice according to the treatment received (*p*-values ranging from 0.001 to 0.003). Indeed, from T1 to T27, mice from the control, vehicle and BaP treatment groups clustered mainly into their own groups (Fig. 2).

**Figure 3:**
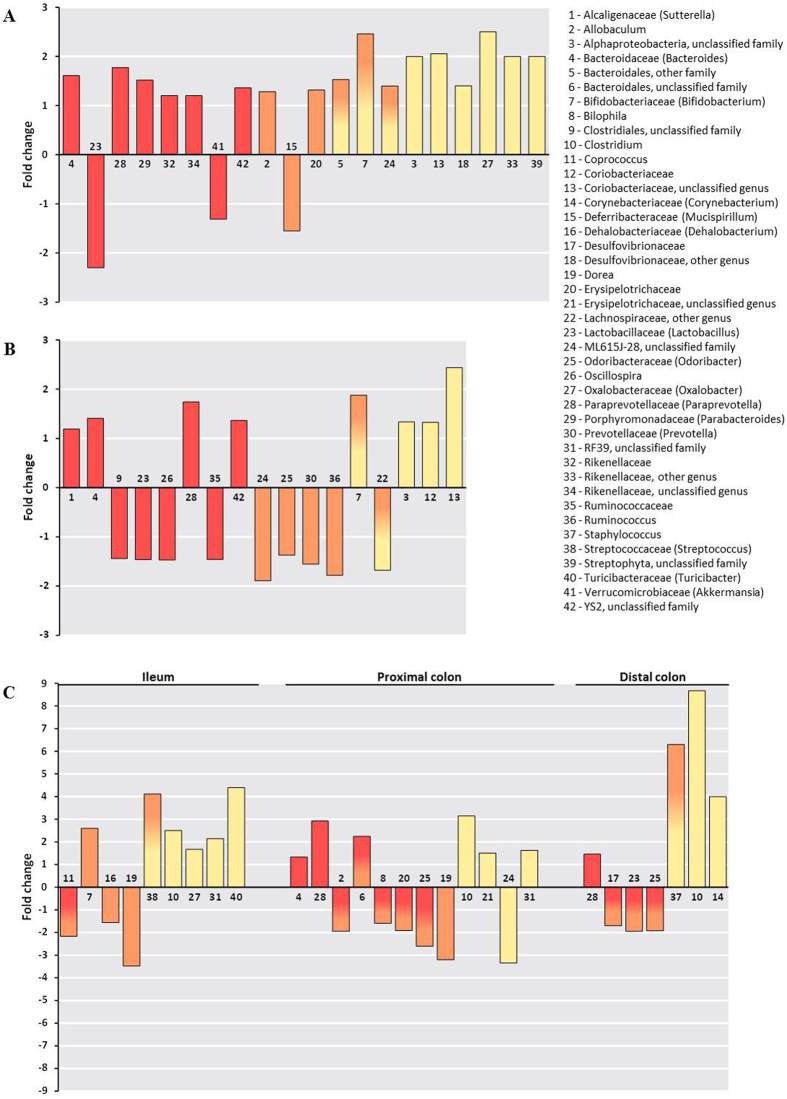
Fold changes and taxa assignments of significantly perturbed faecal and mucosa-associated bacteria due to BaP oral exposure. (**A**) Faecal microbiota in the BaP group at T0 versus T27. (**B**) Faecal microbiota at T27 in the BaP group versus the control group. (**C**) Mucosa-associated microbiota in the BaP group versus the control group. Taxa in yellow were considered as rare members of the BCC (<0.1% in relative abundance), whereas those in red were dominant members (>1% in relative abundance). The colour gradient from red to orange indicates taxa that were initially dominant and then declined and were considered to be common (between 0.1% and 1%). The gradient from yellow to orange shows shifts from rare to common taxa. Significant variations of the bacterial population abundance were assessed using the Mann-Whitney test (*p-value* < 0.05).

Whereas some bacterial taxa were recurrent for all sampling dates in all groups as dominants (e.g., *Bacteroides*, *Paraprevotella* and *Lactobacillus*) or rare (e.g., *Desulfovibrio* and members of *Coriobacteriaceae*), other taxa occurred at only a few dates as belonging to one of these two categories (*Bifidobacterium* and *Coprococcus*) (Table S2). For some of the dominant populations, their abundance decreased below the threshold of 1%, excluding them from the dominant populations (such as members of *Bacteroidales*) without nevertheless becoming rare. Thus, at the end of the experiment, the relative abundance of 15 families and 18 genera was significantly impacted by the oral administration of BaP. Among dominant taxa, the abundances of the Verrucomicrobia and Bacteroidetes phyla significantly decreased and increased, respectively. More precisely, some bacterial families such as *Bacteroidaceae* (*Bacteroides*), *Porphyromonadaceae* (*Parabacteroides*) and *Paraprevotellaceae* (*Paraprevotella*) showed a significant increase in the 16S rRNA relative gene abundance at the end of the BaP treatment (T27), whereas the relative abundance of *Lactobacillaceae* (*Lactobacillus*) and *Verrucomicrobiaceae* (a family exclusively represented by *Akkermansia muciniphila*) decreased (Figs 3A and S4). In the BaP group, 104 significant variations (from phylum to genus) were observed over the course of the experiment, as for Proteobacteria (decrease at T1 and T14 then increase at T21 and T27) (Fig. S4). Similarly, *Verrucomicrobiaceae* (*A. muciniphila*) displayed a significant decrease at T7, followed by an increase at T21 and T27 (Figs S2 and S4). An increase of *Verrucomicrobiaceae* occurred at T1 in the BaP treatment group compared with the control group (Figs S2 and S4). At the end of the experiment involving the oral administration of BaP (T27), the proportions of *Bacteroidaceae* (*Bacteroides*), *Paraprevotellaceae* (*Paraprevotella*) and *Alcaligenaceae* increased, whereas the proportions of *Lactobacillaceae* (*Lactobacillus*) and *Ruminococcaceae* (*Oscillospira*) decreased (Figs 3B and S4).

Modifications of faecal BCC were also observed for both common and rare members (Table S2). Indeed, the relative abundance of *Allobaculum* and *Mucispirillum* increased and decreased, respectively, at the end of the BaP treatment period for common members (Figs 3A and S4). Among rare taxa, the *Actinobacteria* class and some members of *Coriobacteriaceae*, *Rikenellaceae*, and *Desulfovibrionaceae* families presented higher abundances at the end of the experiment (Fig. S4). Some of these taxa, such as *Bifidobacterium* and members of the *Bacteroidales* (other family), increased and became common (Table S2 and Fig. S4). Compared with the control group, the *Actinobacteria* class and some members of the *Coriobacteriaceae* abundances were significantly increased in the BaP treatment group after 28 days of oral exposure (Fig. 3B and Fig. S4).

### Effects of BaP on mucosa-associated BCC

**Figure 4:**
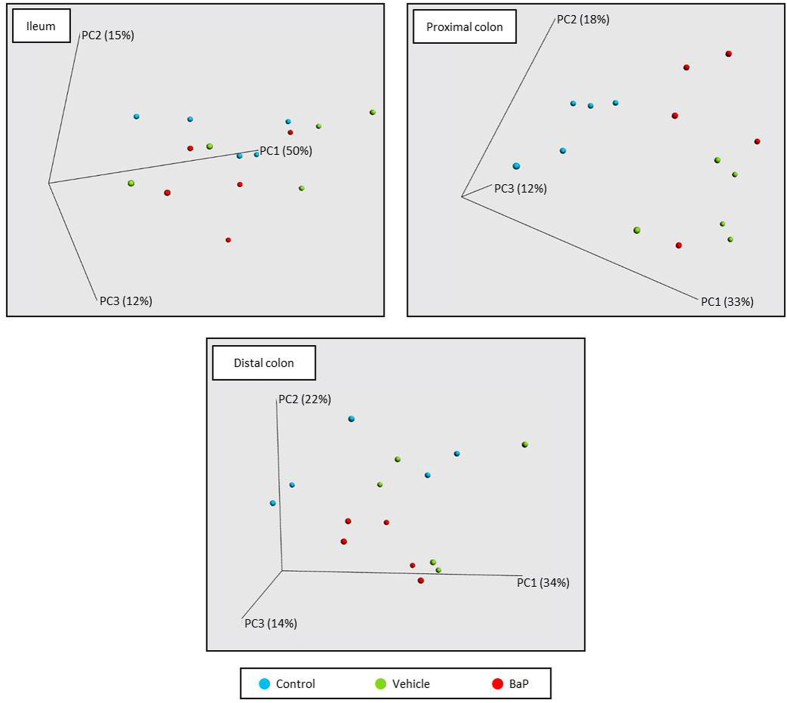
Mucosa-associated bacterial patterns of ileum, proximal and distal colon differentiated by principal coordinate analysis (PCoA) on a weighted UniFrac distance matrix for control, vehicle and BaP-treated mice. Control, vehicle and BaP-treated mice are represented in blue, green and red, respectively. The R-value from ANOSIM were −0.017 (*p*-value = 0.460), 0.547 (*p*-value = 0.001), and 0.146 (*p*-value = 0.075) respectively for ileum, proximal and distal colon.

Beta-diversity analyses revealed differences between the BCC of different groups, and multivariate analyses revealed that the mucosa-associated microbiota clustered according to gut localization (Fig. S6). PCoA plots also revealed a separation between control and BaP groups and more especially for the colonic segments (Fig. 4).

Alterations in the mucosa-associated microbiota profiles were more evident at the bacterial genus level with 31 significant changed genera. As observed for the faecal microbiota, the abundance of some dominant members significantly decreased after BaP treatment. Thus, compared to the control group, decreased relative abundances were observed for *Coprococcus* (*Lachnospiraceae*) in the ileal segment, *Allobaculum* (*Erysipelotrichaceae*) and *Bilophila* (*Desulfovibrionaceae*) in the proximal colon, and *Lactobacillus* and *Desulfovibrionaceae* in the distal colon (Fig. 3C). *Paraprevotella* and *Bacteroides* (only in the proximal colon) were increased in the colonic mucosa. In the ileal segment, *Bifidobacterium* and *Dehalobacterium*, which were defined as common taxa, increased and decreased, respectively. A lower relative abundance of *Dorea* (*Lachnospiraceae*) was also observed in both the ileum and proximal colon. Among the rare taxa, compared to the control group, an increase of *Clostridium* was detected in all intestinal segments studied, whereas *Turicibacter* and *Oxalobacter* presented a greater abundance only in the ileum (Fig. 3C). The *Corynebacterium* genus was also increased in the distal colon in the BaP treatment group. Some rare bacterial families (*Bacilliaceae* and *Streptococcaceae*) were increased in the ileal mucosa-associated microbiota for the BaP treatment group and became common taxa (Table S3).

## Discussion

The composition of the gut microbiota can have a profound impact on human health. Emerging evidence has suggested that environmental factors, especially pollution, may be associated with several pathologies. Because links between gut microbiota and diseases and between pollution and pathologies have been previously documented, we investigated the potential effects of BaP subchronic oral exposure both on the intestinal epithelium of different parts of the gastrointestinal tract and on the gut bacterial structure in C57BL/6 mice.

As showed in previous analyses^17^, a lower richness and diversity have been observed for the mucosa-associated microbiota compare to the faecal samples. However, these differences can be explained in part by the great disparity in the sequencing depth between these two types of microbiota samples. Despite that BaP is a molecule characterized by toxic properties and for which modifications of bacterial communities from different environments were observed (e.g., Sawluski *et al*.^18^), no significant impact of BaP was found on the bacterial richness or diversity of either stools or intestinal mucosa tissues in mice, as it was also observed for other environmental pollutants such as PCB or heavy metals^6^,^19^.

If no effect was shown on alpha-diversity, the data underlined that BaP oral exposure induced significant shifts in the composition and relative abundance of stool and mucosa-associated bacterial communities in mice (Mann-Whitney test *p*-value < 0.05). Although the physiological and anatomical structures of humans and mice are quite similar, and most of the mechanisms of microbiota-host interactions are shared between these species, some key differences exist between murine models and humans. For instance, although 85% of the bacterial species found in mice are not present in humans^20^, similar trends have been observed in the gut microbiota composition^21^. Thus, in our work, the Firmicutes and Bacteroidetes phyla predominated the faecal and mucosa-associated BCC, which was consistent with the results of other studies^22^,^23^. Moreover, although bacterial populations displayed a broad diversity and taxonomic composition that could varied during the kinetics of each experimental mouse group and between treatments, the general structure of the bacterial assembly remained the same. Indeed, both the faecal and mucosa-associated BCC were dominated by a small number of taxa.

Oral exposure to BaP appeared to elicit environmental stress mainly on the bacterial family and genus levels. For example, no decrease in Firmicutes was detected following BaP oral exposure, whereas *Lactobacillaceae,* and more particularly *Lactobacillus,* decreased at the end of the experimental period for faecal BCC (T27). *Lactobacillaceae* are reduced in ulcerative colitis patients^24^ and also in mice that have been exposed to heavy metals such as cadmium^5^. Other bacterial taxa that are also recognized as beneficial and that decrease in abundance in inflammatory diseases, such as *Mucispirillum* and *Ruminococcaceae*, were selectively affected during the course of BaP administration and at the end of the experimental period for faecal analyses. *Mucispirillum,* which is a mucolytic genus, plays an important role in regulating host gene expression and cellular functions^25^. *Ruminococcaceae*, which produce short chain fatty acids (SCFAs) (acetate) that promote intestinal and systemic health, are depleted in Crohn’s disease^26^. Following the first BaP oral administration (T1), an increase in abundance of beneficial bacterium *A. muciniphila* was observed. The abilities of this bacterium to degrade mucus and to produce propionate which notably stimulates the immune system and the intestinal barrier functions, have been demonstrated^27^. The bloom of *A. muciniphila* one day after the first administration of BaP may suggest a protective effect on the intestinal epithelium. However, this protective effect appeared to be temporary, because a global decrease in the relative abundance of this bacterium was observed during exposure to BaP. These depletions of different beneficial bacterial taxa seem to occur with the onset of a pro-inflammatory intestinal environment, in which members of the *Porphyromonadaceae*, *Paraprevotellaceae*, and *Alcaligenaceae* families, which are known to be associated with inflammatory states, increase in abundance^23^,^28^,^29^. *Allobaculum,* which belongs to the *Erysipelotrichaceae* family, was also increased. However, the role of this bacterial genus remains still unclear: Everard *et al*.^22^ have identified *Allobaculum* as being potentially beneficial for host physiology, whereas Wei *et al*.^30^ have found that this genus is increased in the faeces of rats developing precancerous mucosal lesions.

The rare members of the faecal bacterial communities also underwent temporal changes, like the dominant members. Moreover they can play an important role in the physiology and homeostasis of the gastrointestinal tract. Indeed if they are low contributors to the total community abundance, these members can represent substantial contributors to the functioning of the ecosystem^16^. In fact, rare members can rapidly increase their presence in response to environmental changes by acting as an active microbial seed bank with a pool of ecological potential. The increase of some rare bacterial taxa that are considered to be harmful to the gut would not be surprising. Thus, BaP oral exposure could lead to the development of a favourable environment for populations that are non-beneficial to the host. These populations include *Desulfovibrionaceae* members, whose increase has previously been related to the occurrence of host inflammation^31^ and colitis in a murine model^32^. It has also been suggested that *Desulfovibrionaceae* members could produce endotoxins^33^ and toxic sulfur^34^, which has the capacity to induce inflammation^35^. To validate these hypotheses, endotoxins and LPS productions should be monitored in future studies. We also observed an increase in rare taxa that are considered to be beneficial for the functioning of the intestinal tract, such as *Bifidobacterium* at the end of BaP oral exposure. This lactic bacterial genus has been particularly associated with IBD remission^32^. In context of the bacterial community functioning in ecosystems, integrating especially the concepts of stability and resistance, the *Bifidobacterium* bloom could underline the important role of rare taxa in intestinal homeostasis. Indeed the recruitment of beneficial bacteria can counteract the negative effects of BaP and the higher abundance of pro-inflammatory populations. Their increase could therefore contribute to maintain the gut ecosystem stability *via* resistance phenomenon. Nevertheless, this remains hypothetical and needs to be confirmed in subsequent studies. The capacity of a community to accommodate to environmental changes can be realized by adjusting the overall performance of the present taxa. In the gut, metabolic plasticity or functional redundancy may also be present at the community level, indicating that a similar functional role can be performed by several taxa in the community.

Several differences have been highlighted between the BCC from stools and the intestinal mucosa. The intestinal epithelium plays an important role as barrier that prevents the entry of bacteria and the passage of microbial components that could drive inflammation. Thus, the mucus layer, which is secreted by the intestinal epithelium, acts as the first physical line of defence and with mucosa-associated bacteria, forms a specific environment in the gut. The alteration of any of these two parameters may play a crucial role in the development of inflammation, and consequently, in the development of intestinal pathologies. Spatial variations were observed in mucosal BCC at family and genus level at the end of the BaP treatment. For all gut segments studied, a decrease of beneficial bacteria was consistent with an increase of pro-inflammatory taxa and a moderate grade of inflammation of the intestinal mucosa. Thus, the depletion of *Coprococcus* (*Lachnospiraceae* family), a butyrate-producer, in the ileum of BaP-treated mice has also been shown in IBD^36^. As *Ruminococcaceae*, *Lachnospiraceae* participate in the carbohydrate fermentation of SCFAs in the human intestine^37^. A decrease in SCFA production linked to a reduction of these bacterial populations may damage the barrier function of the intestinal epithelium and increased susceptibility to inflammation^38^. The decrease of *Lactobacillaceae* in the distal colon in BaP-treated mice suggests a negative impact of the pollutant. Indeed, the immunomodulatory functions of *Lactobacillaceae* have been highlighted in the colon, revealing their potential as probiotics for treating inflammation in IBD and colitis in a murine model^39^,^40^. In the ileum, the reduction of these different dominant bacterial taxa with anti-inflammatory effects in the BaP treatment group coincided with an increase of dominant and rare bacterial taxa that may be pro-inflammatory, such as *Paraprevotellaceae* (dominant) and *Turicibacter* (rare). For instance, *Turicibacter* has been found at high levels in the ileal pouch of an ulcerative colitis patient^41^. Following BaP oral exposure, the bloom of the *Clostridium* genus among the rare taxa was observed within the 3 intestinal segments. The role of *Clostridium* could not be defined in our study even though they may take advantage of the alteration of the intestinal epithelium to synthesize potentially toxic metabolic products (e.g., phenols and p-cresols)^42^. Moreover, *Clostridium* have been shown to increase in abundance in patients with ulcerative colitis^24^. However, we should also note that some *Clostridium* species produce butyrate^43^. An increase in *Clostridium* species may contribute to the resistance to the pro-inflammatory environment resulting from oral exposure to BaP.

Chronic oral exposure to BaP caused more pronounced inflammation in the ileal segment than in the colon. This result was concordant with the results of Allais *et al*.^44^, who emphasized that unlike the ileum, the distal colon does not express inflammatory factors in mice subjected to chronic smoke exposure. Similarly, the colon appeared to respond differently than the small intestine to particulate matter^45^. Mucus layer thickness and bacterial density, which are higher in the colon than in the ileum, could contribute to the reduced effect of BaP on the colonic segment. Besides, the small intestine is a known target organ for BaP toxicity, and DNA adduct formation has been demonstrated in the small intestine of mice that were orally exposed to BaP^46^. The small intestine has been previously shown to be the first segment of the gut to engage in the P450 enzyme-mediated metabolism of absorbed xenobiotics^47^. The high activity of P450 enzymes in this gut segment could therefore protect the colon against oral exposure to BaP^48^. Moreover, during the investigation of both faecal and mucosa-associated BCC, some bacterial taxa may be identified as actors of xenobiotic degradation. Thus, for *Coriobacteriaceae* members, that increased at the end of the oral exposure to BaP, a strong correlation was found between them and the activity of Cyp3a11, one of the most active cytochrome P450 enzymes involved in drug metabolism in mice^49^. Therefore, some members of this family may intervene in the degradation of BaP. An increase of *Alcaligenaceae* was also observed in the faecal samples. *Alcaligenaceae* belongs to the *Burkholderiales* order, for which numerous members have been identified as PAH degraders^50^. In the same way, the bacterial genera specifically detected in the BaP group, such as *Bacillus* in the ileum and *Acinetobacter* in the proximal colon, include species that have been identified as producers of biosurfactants and degraders of PAHs, including BaP^50^. Additionally, it was also suggested that gut microbiota is able to metabolize PAHs into estrogenic metabolites^51^. These results support the potential role of gut microbiota in BaP degradation.

To conclude, subchronic oral exposure to BaP induced moderate inflammation primarily in the ileal mucosa and altered the relative abundance of both faecal and mucosa-associated microbiota. At the beginning of the experiment, the gut microbiota may have had a transient protecting effect against the pro-inflammatory proprieties of BaP, especially through the increase of mucolytic taxa. However, after 28 days of oral exposure, BaP led to a pro-inflammatory intestinal environment, with an increase in the pro-inflammatory bacterial taxa and a decrease in the anti-inflammatory bacterial taxa. Even if this study remains descriptive, it is the first work to assess the potential effects of BaP on gut microbiota, especially to analyse both faecal microbiota at different time points and mucosa-associated bacteria at different localisations of the gut. This work establishes a basis for more complex and functional studies. Indeed, to confirm the relationship between gut microbiota shifted by BaP oral exposure and the intestinal functions, gene expression modulation from host and microbial communities, using RNAseq approaches, should be monitored. Such studies will also aim to confirm or refute that under the conditions of genetic susceptibility and in association with other environmental factors, oral exposure to BaP could trigger and/or accelerate the development of inflammatory pathologies such as IBD, autoimmune disorders and colorectal cancers.

## Methods

### Animals

Animal protocols were approved by the Committee for Research and Ethical Issues of the CEMEAA (Comité d’Ethique en Matière d’Expérimentation Animale Auvergne, France) (CE73-12) and were carried out in accordance with the international directive 86/609/CEE. The animals were treated humanely and with regard for the alleviation of suffering.

Five-week-old male C57BL/6 mice weighing 20–25 g were purchased from the Charles River Laboratory (Wilmington, MA, USA) and were allowed a 14-day acclimatization period prior to the start of the study. They were kept in standard housing conditions with access to rodent chow and water *ad libitum*.

### Experimental design

Mice were randomly divided into 3 groups, and the cages were protected with filters to avoid contamination with faecal pellets. Group 1 (control group; n = 10) was given BaP-free physiological saline solution, group 2 (vehicle group; n = 8) was fed with sunflower seed oil (Sigma-Aldrich, Saint-Quentin Fallavier, France), and group 3 (BaP group; n = 11) was supplied with BaP (96% purity, Sigma-Aldrich, Saint-Quentin Fallavier, France) dissolved in sunflower seed oil at a concentration of 50 mg/kg of body weight (BW) (corresponding to BaP-inducing genotoxic and carcinogenic effects, as determined in previous studies (e.g., Malik *et al*.^52^). All treatments were performed *via* oral gavage (10 mL/kg of BW) for 28 consecutive days in order to simulate a subchronic exposure (e.g. Labib *et al*.^53^). The weight of the mice was monitored every week during the BaP oral exposure. Stools from each mouse were collected at day 0, 1 (24 hours after the first administration of BaP), 7, 14, 21, and 27 and stored at −80 °C until DNA extraction. At the end of the experiment the mice were sacrificed. The ileum and colon were immediately harvested and washed in phosphate-buffered saline. Two cm sections of the ileum (upstream of the caecum) and 1.5-cm sections of the proximal and distal colon were collected and frozen at −80 °C until DNA extraction to access to the mucosa-associated bacteria. The remaining ileum and colon were kept for histological analysis.

### Histological grading of intestinal inflammation

After the mice were sacrificed, the remaining ileum and colon segments were prepared as Swiss rolled, fixed in 4% paraformaldehyde for 24 hours, embedded in paraffin, cut into 5 μm slices, and stained with hematoxylin and eosin. Histological scoring of inflammation was graded in a blinded fashion by a pathologist according to 4 criteria, each of them ranging from 0–3 (Table S4). The histological score corresponds to the sum of all of the criteria, with a possible maximum of 12.

### Microbiota analysis by 16S rRNA gene sequencing

Genomic DNA (gDNA) was extracted from faecal and mucosal samples by using QIAamp^®^ DNA Stool Mini Kit (Qiagen, Courtabœuf, France) and NucleoSpin^®^ Tissue Kit (Macherey-Nagel, Hoerdt, France), respectively, following the manufacturer’s instructions. Five mice per group were chosen for 16S rRNA gene analysis by high-throughput sequencing (Illumina) based on both the histological score and the DNA quantity and quality. The V4 variable region of the 16S rRNA genes was amplified by PCR using universal primers F515 (5′-GTGCCAGCMGCCGCGGTAA-3′) and R806 (5′-GGACTACHVGGGTWTCTAAT-3′)^54^. The DNA library was constructed following TruSeq DNA library preparation protocol (Illumina, San Diego, CA, USA). Paired-end sequencing (2 × 250 bp) was performed at MR DNA (www.mrdnalab.com, Shallowater, TX, USA) on a MiSeq platform.

### High-throughput sequencing data analysis

Paired-end reads were joined using fastq-join from the ea-utils software package^55^. Sequences were analysed using the open source software package Quantitative Insights Into Microbial Ecology (QIIME v. 1.8.0)^56^. After demultiplexing and quality filtering of sequences, chimeras were detected and removed using USEARCH 6.1 (http://www.drive5.com/usearch). The resulting high quality sequences were processed to generate OTUs with a 97% similarity threshold that were then taxonomically assigned on Greengenes database using UCLUST (http://www.drive5.com/uclust).

A variable number of sequences was obtained per sample (range [20,641–231,849] and [5,804–418,919] for stool and mucosa samples, respectively). Therefore, for fair comparison, the sequence number of each sample was randomly normalized to the same sequencing depth (20,500 sequences per stool sample and 5,800 sequences per mucosal sample). The alpha (Chao 1, Shannon’s diversity index, phylogenetic diversity (PD)) and beta-diversity (Principal Coordinate Analyses (PCoA) on a weighted Unifrac distance matrix analyses) were performed using QIIME.

### Statistical Analysis

Statistical analyses of the histological scores were performed using a Kruskal-Wallis nonparametric test with GraphPad Prism 5 software (San Diego, CA, USA) with a statistical significance of *p* < 0.05. Analysis of Similarity (ANOSIM) was applied to test for significant differences between sample groupings. Significant variations in bacterial population richness, diversity and abundance were assessed using Mann-Whitney (*p* < 0.05) and Kruskal-Wallis tests.

### Sequence data accession number

All 16S rRNA gene sequence data are available through the Sequence Read Archive under accession no. SRX1430490.

## Additional Information

**How to cite this article**: Ribière, C. *et al*. Oral exposure to environmental pollutant benzo[a]pyrene impacts the intestinal epithelium and induces gut microbial shifts in murine model. *Sci. Rep.* **6**, 31027; doi: 10.1038/srep31027 (2016).

## Supplementary Material

Supplementary Information
