# Supplementary material for: Oral exposure to environmental pollutant benzo[a]pyrene impacts the intestinal epithelium and induces gut microbial shifts in murine model
Source: Sci Rep. 2016 Aug 9;6:31027. doi: 10.1038/srep31027 (PMC4977522; doi:10.1038/srep31027)
Supplement: Supplementary Information [file srep31027-s1.pdf]

Oral exposure to environmental pollutant benzo[a]pyrene impacts the intestinal epithelium and induces gut microbial shifts in murine model.

Supplementary Information

Céline Ribière, Pierre Peyret, Nicolas Parisot, Claude Darcha, Pierre J. Déchelotte, Nicolas Barnich, Eric Peyretaillade *†, Delphine Boucher †

† These authors contributed equally to this work.

Table of contents:

Table S1. Richness (OTU number, Chao1) and diversity (Shannon, Phylogenetic Diversity) values for fecal and mucosa-associated bacteria of different mouse groups (control, vehicle, BaP).

Table S2. Shifts in faecal bacterial community composition.

Table S3. Shifts in mucosa-associated bacterial community composition.

Table S4: Histological scoring parameters.

Fig S1. The score of each criterion used in the combined histological scoring of inflammation for the ileum and colon of mice.

Fig S2. Faecal microbiota composition profiles at the family level in mice for control (a), vehicle (b) and BaP-treated groups (c) revealed by 16S rRNA gene sequencing.

Fig S3. Shared and specific bacterial genera detected in control, vehicle and BaP-treated groups for faecal (a) and mucosa-associated microbiota in the ileum (b), proximal colon (c) and distal colon (d).

Fig S4. Mucosa-associated microbiota composition profiles at the family level in the ileum (a), proximal colon (b) and distal colon (c) in mice revealed by 16S rRNA gene sequencing.

Fig S5. Mucosa-associated bacteria of the ileum, proximal colon and distal colon for control, vehicle and BaP-treated mice differentiated by principal coordinate analysis (PCoA) on a weighted UniFrac distance matrix.

Table S1. Richness (OTU number, Chao1) and diversity (Shannon, Phylogenetic Diversity) values for fecal and mucosa-associated bacteria of different mouse groups (control, vehicle, BaP). The values are given as the means ± SD. The operational taxonomic units (OTUs) were defined with 97% sequence identity. Calculations were made based on rarefied OTU tables at 20,500 and 5,800 sequences for fecal and mucosal samples, respectively.

	**Faecal microbiota**						**Mucosal microbiota**		
Index	T0	T1	T7	T14	T21	T27	Ileum	Proximal colon	Distal colon
**OTUs**									
Control	4 174 ± 246	3 866 ± 583	4 403 ± 82	4 036 ± 530	4 244 ± 162	4 344 ± 96	1 067 ± 193	1 107 ± 36	1 155 ± 288
Vehicle	4 042 ± 290	4 050 ± 138	4 183 ± 440	4 073 ± 198	4 222 ± 269	4 139 ± 177	906 ± 300	985 ± 196	918 ± 58
BaP	4 081 ± 219	3 830 ± 269	4 090 ± 417	4 401 ± 110	4 312 ± 209	3 988 ± 187	912 ± 225	1 224 ± 167	978 ± 42
**Chao1**									
Control	15 082.4 ± 1 451.3	14 354.3 ± 2 086.6	15 960.8 ± 466.8	15 162.3 ± 1 618.9	15 426.6 ± 770.1	15 831.8 ± 516.4	4 345.3 ± 756.5	4 433.6 ± 590.8	4 585.1 ± 1 155.7
Vehicle	15 245.6 ± 1 082.1	14 575.3 ± 728 5	15 413.1 ± 1 499.9	15 158.3 ± 936.9	15 655.8 ± 1 232.2	15 287.8 ± 834.0	3 708.0 ± 1 643.8	3 932.3 ± 829.1	3 900.3 ± 619.4
BaP	14 455.9 ± 1 130.9	13 946.5 ± 953.9	14 389.2 ± 1 410.2	15 940.5 ± 406.0	16 139.6 ± 826.0	14 343.1 ± 983.6	3 534.4 ± 940.9	5 054.4 ± 958.1	3 867.7 ± 303.4
**Shannon**									
Control	8.52 ± 0.25	8.44 ± 0.35	8.87 ± 0.10	8.65 ± 0.36	8.57 ± 0.19	8.71 ± 0.11	7.07 ± 0.54	7.61 ± 0.14	7.58 ± 0.45
Vehicle	8.57 ± 0.18	8.49 ± 0.14	8.55 ± 0.37	8.36 ± 0.24	8.48 ± 0.33	8.45 ± 0.26	6.05 ± 1.09	6.97 ± 0.59	7.06 ± 0.20
BaP	8.35 ± 0.25	8.05 ± 0.18	8.65 ± 0.31	8.84 ± 0.11	8.68 ± 0.18	8.35 ± 0.22	6.35 ± 0.93	7.65 ± 0.47	7.31 ± 0.25
**PD**									
Control	365.9 ± 18.2	348.9 ± 38.7	378.8 ± 8.1	358.3 ± 34.9	372.7 ± 8.2	378.4 ± 5.2	122.8 ± 15.8	125.6 ± 3.2	130.5 ± 24.4
Vehicle	360.6 ± 15.5	355.2 ± 8.7	364.6 ± 25.0	361.7 ± 13.6	368.1 ± 15.1	364.2 ± 12.3	108.1 ± 24.2	116.3 ± 15.5	109.9 ± 4.1
BaP	360.5 ± 13.4	343.7 ± 18.5	360.9 ± 25.7	379.9 ± 7.5	375.7 ± 12.6	356.3 ± 10.1	108.4 ± 18.4	133.9 ± 12.9	115.8 ± 3.2

Table S2. Shifts in faecal bacterial community composition. The colors represent the relative abundance of different bacterial genera detected in fecal samples. Genera highlighted in yellow were considered as rare members of the BCC (<0.1% in relative abundance), those in red were dominant members (>1% in relative abundance), and those in orange were common taxa (between 0.1% and 1% in relative abundance).

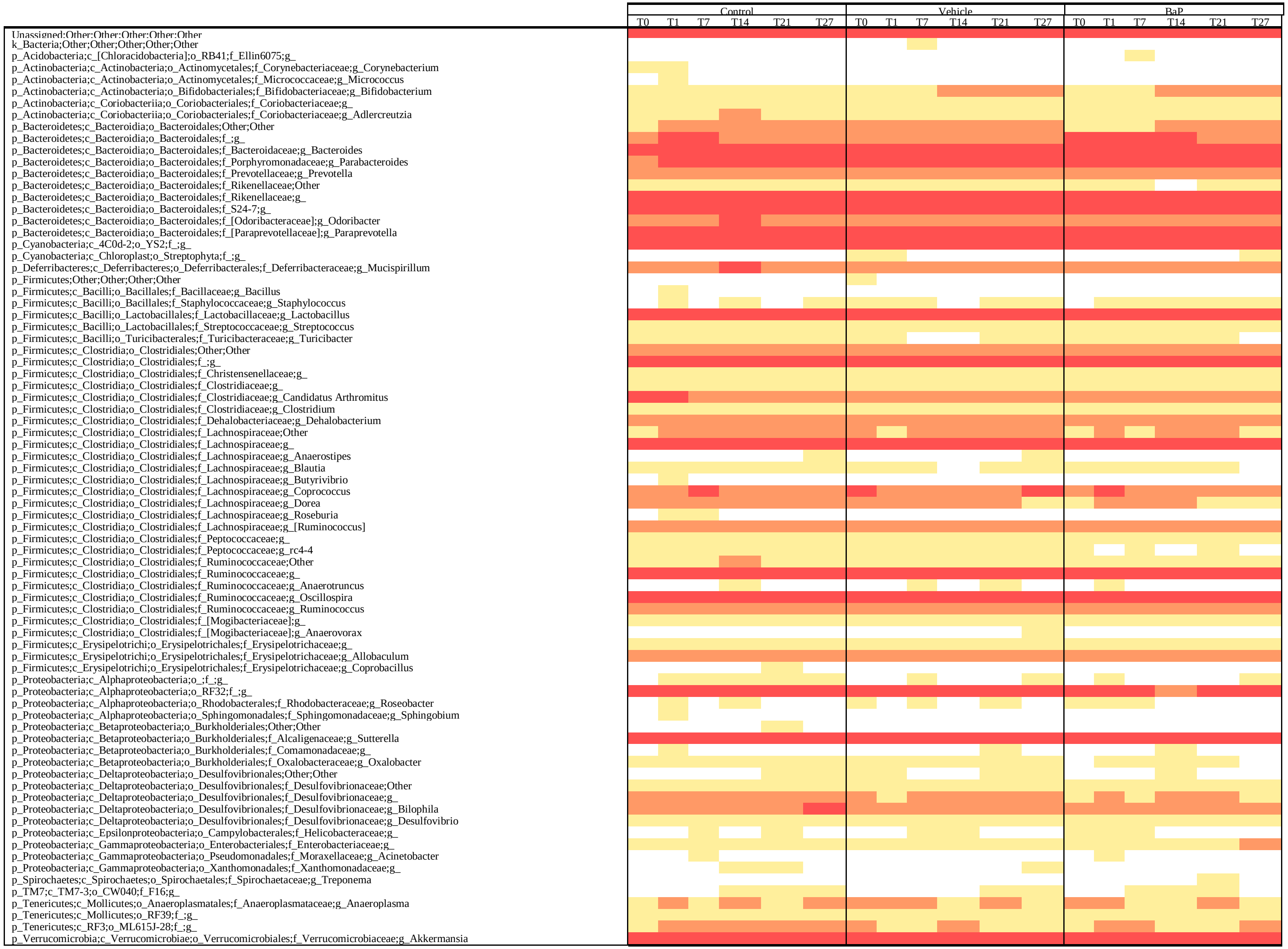

Table S3. Shifts in mucosa-associated bacterial community composition. The colors represent the relative abundance of different bacterial genera detected in mucosal samples. Genera highlighted in yellow were considered as rare members of the BCC (<0.1% in relative abundance), those in red were dominant members (>1% in relative abundance), and those in orange were common taxa (between 0.1% and 1% in relative abundance).

	Ileum			Proximal colon			Distal colon		
	Control	Vehicle	BaP	Control	Vehicle	BaP	Control	Vehicle	BaP
Unassigned;Other;Other;Other;Other;Other									
p_Acidobacteria;c_[Chloracidobacteria];o_RB41;f_Ellin6075;g_									
p_Actinobacteria;c_Actinobacteria;o_Actinomycetales;f_Corynebacteriaceae;g_Corynebacterium									
p_Actinobacteria;c_Actinobacteria;o_Actinomycetales;f_Micrococcaceae;g_Micrococcus									
p_Actinobacteria;c_Actinobacteria;o_Bifidobacteriales;f_Bifidobacteriaceae;g_Bifidobacterium									
p_Actinobacteria;c_Coriobacteriia;o_Coriobacteriales;f_Coriobacteriaceae;g_									
p_Actinobacteria;c_Coriobacteriia;o_Coriobacteriales;f_Coriobacteriaceae;g_Adlercreutzia									
p_Bacteroidetes;c_Bacteroidia;o_Bacteroidales;Other;Other									
p_Bacteroidetes;c_Bacteroidia;o_Bacteroidales;f_;g_									
p_Bacteroidetes;c_Bacteroidia;o_Bacteroidales;f_Bacteroidaceae;g_Bacteroides									
p_Bacteroidetes;c_Bacteroidia;o_Bacteroidales;f_Porphyromonadaceae;g_Parabacteroides									
p_Bacteroidetes;c_Bacteroidia;o_Bacteroidales;f_Prevotellaceae;g_Prevotella									
p_Bacteroidetes;c_Bacteroidia;o_Bacteroidales;f_Rikenellaceae;Other									
p_Bacteroidetes;c_Bacteroidia;o_Bacteroidales;f_Rikenellaceae;g_									
p_Bacteroidetes;c_Bacteroidia;o_Bacteroidales;f_S24-7;g_									
p_Bacteroidetes;c_Bacteroidia;o_Bacteroidales;f_[Odoribacteraceae];g_Odoribacter									
p_Bacteroidetes;c_Bacteroidia;o_Bacteroidales;f_[Paraprevotellaceae];g_Paraprevotella									
p_Cyanobacteria;c_4C0d-2;o_YS2;f_;g_									
p_Cyanobacteria;c_Chloroplast;o_Streptophyta;f_;g_									
p_Deferribacteres;c_Deferribacteres;o_Deferribacterales;f_Deferribacteraceae;g_Mucispirillum									
p_Firmicutes;c_Bacilli;o_Bacillales;f_Bacillaceae;g_Bacillus									
p_Firmicutes;c_Bacilli;o_Bacillales;f_Staphylococcaceae;g_Staphylococcus									
p_Firmicutes;c_Bacilli;o_Lactobacillales;f_Lactobacillaceae;g_Lactobacillus									
p_Firmicutes;c_Bacilli;o_Lactobacillales;f_Streptococcaceae;g_Streptococcus									
p_Firmicutes;c_Bacilli;o_Turicibacterales;f_Turicibacteraceae;g_Turicibacter									
p_Firmicutes;c_Clostridia;o_Clostridiales;Other;Other									
p_Firmicutes;c_Clostridia;o_Clostridiales;f_;g_									
p_Firmicutes;c_Clostridia;o_Clostridiales;f_Christensenellaceae;g_									
p_Firmicutes;c_Clostridia;o_Clostridiales;f_Clostridiaceae;g_									
p_Firmicutes;c_Clostridia;o_Clostridiales;f_Clostridiaceae;g_Candidatus Arthromitus									
p_Firmicutes;c_Clostridia;o_Clostridiales;f_Clostridiaceae;g_Clostridium									
p_Firmicutes;c_Clostridia;o_Clostridiales;f_Dehalobacteriaceae;g_Dehalobacterium									
p_Firmicutes;c_Clostridia;o_Clostridiales;f_Lachnospiraceae;Other									
p_Firmicutes;c_Clostridia;o_Clostridiales;f_Lachnospiraceae;g_									
p_Firmicutes;c_Clostridia;o_Clostridiales;f_Lachnospiraceae;g_Blautia									
p_Firmicutes;c_Clostridia;o_Clostridiales;f_Lachnospiraceae;g_Coprococcus									
p_Firmicutes;c_Clostridia;o_Clostridiales;f_Lachnospiraceae;g_Dorea									
p_Firmicutes;c_Clostridia;o_Clostridiales;f_Lachnospiraceae;g_[Ruminococcus]									
p_Firmicutes;c_Clostridia;o_Clostridiales;f_Peptococcaceae;g_									
p_Firmicutes;c_Clostridia;o_Clostridiales;f_Peptococcaceae;g_rc4-4									
p_Firmicutes;c_Clostridia;o_Clostridiales;f_Ruminococcaceae;Other									
p_Firmicutes;c_Clostridia;o_Clostridiales;f_Ruminococcaceae;g_									
p_Firmicutes;c_Clostridia;o_Clostridiales;f_Ruminococcaceae;g_Oscillospira									
p_Firmicutes;c_Clostridia;o_Clostridiales;f_Ruminococcaceae;g_Ruminococcus									
p_Firmicutes;c_Clostridia;o_Clostridiales;f_[Mogibacteriaceae];g_									
p_Firmicutes;c_Erysipelotrichi;o_Erysipelotrichales;f_Erysipelotrichaceae;g_									
p_Firmicutes;c_Erysipelotrichi;o_Erysipelotrichales;f_Erysipelotrichaceae;g_Allobaculum									
p_Proteobacteria;c_Alphaproteobacteria;o_;f_;g_									
p_Proteobacteria;c_Alphaproteobacteria;o_RF32;f_;g_									
p_Proteobacteria;c_Betaproteobacteria;o_Burkholderiales;f_Alcaligenaceae;g_Sutterella									
p_Proteobacteria;c_Betaproteobacteria;o_Burkholderiales;f_Comamonadaceae;g_									
p_Proteobacteria;c_Betaproteobacteria;o_Burkholderiales;f_Oxalobacteraceae;g_Oxalobacter									
p_Proteobacteria;c_Deltaproteobacteria;o_Desulfovibrionales;f_Desulfovibrionaceae;g_									
p_Proteobacteria;c_Deltaproteobacteria;o_Desulfovibrionales;f_Desulfovibrionaceae;g_Bilophila									
p_Proteobacteria;c_Deltaproteobacteria;o_Desulfovibrionales;f_Desulfovibrionaceae;g_Desulfovibrio									
p_Proteobacteria;c_Gammaproteobacteria;o_Enterobacteriales;f_Enterobacteriaceae;g_									
p_Proteobacteria;c_Gammaproteobacteria;o_Pseudomonadales;f_Moraxellaceae;g_Acinetobacter									
p_Proteobacteria;c_Gammaproteobacteria;o_Pseudomonadales;f_Pseudomonadaceae;g_Pseudomonas									
p_Proteobacteria;c_Gammaproteobacteria;o_Xanthomonadales;f_Xanthomonadaceae;g_									
p_Tenericutes;c_Mollicutes;o_Anaeroplasmatales;f_Anaeroplasmataceae;g_Anaeroplasma									
p_Tenericutes;c_Mollicutes;o_RF39;f_;g_									
p_Tenericutes;c_RF3;o_ML615J-28;f_;g_									
p_Verrucomicrobia;c_Verrucomicrobiae;o_Verrucomicrobiales;f_Verrucomicrobiaceae;g_Akkermansia									

Table S4: Histological scoring parameters. The total inflammation score was determined by the sum of each criterion and could reach a maximum of 12.

**Score**	**0**	**1**	**2**	**3**
Infiltration of inflammatory cells (x10 magnification)	Rare inflammatory cells in the lamina propria	Increased number of inflammatory cells, including neutrophils in the lamina propria	Confluence of inflammatory cells extending into submucosa	Transmural extension of the inflammatory cell infiltrate
Infiltration of epithelium by polynuclear cells (x20 magnification)	No infiltration	Few inside the crypt	Lot of inside the crypt	Cryptic abscess
Severity of epithelial damage	Absence of mucosal damage	Lymphoepithelial lesions	Mucosal erosion/ulceration	Extensive mucosal damage and extension through deeper structure of the bowel wall
Surface of epithelial damage	Normal	Focal	Multifocal	Wide

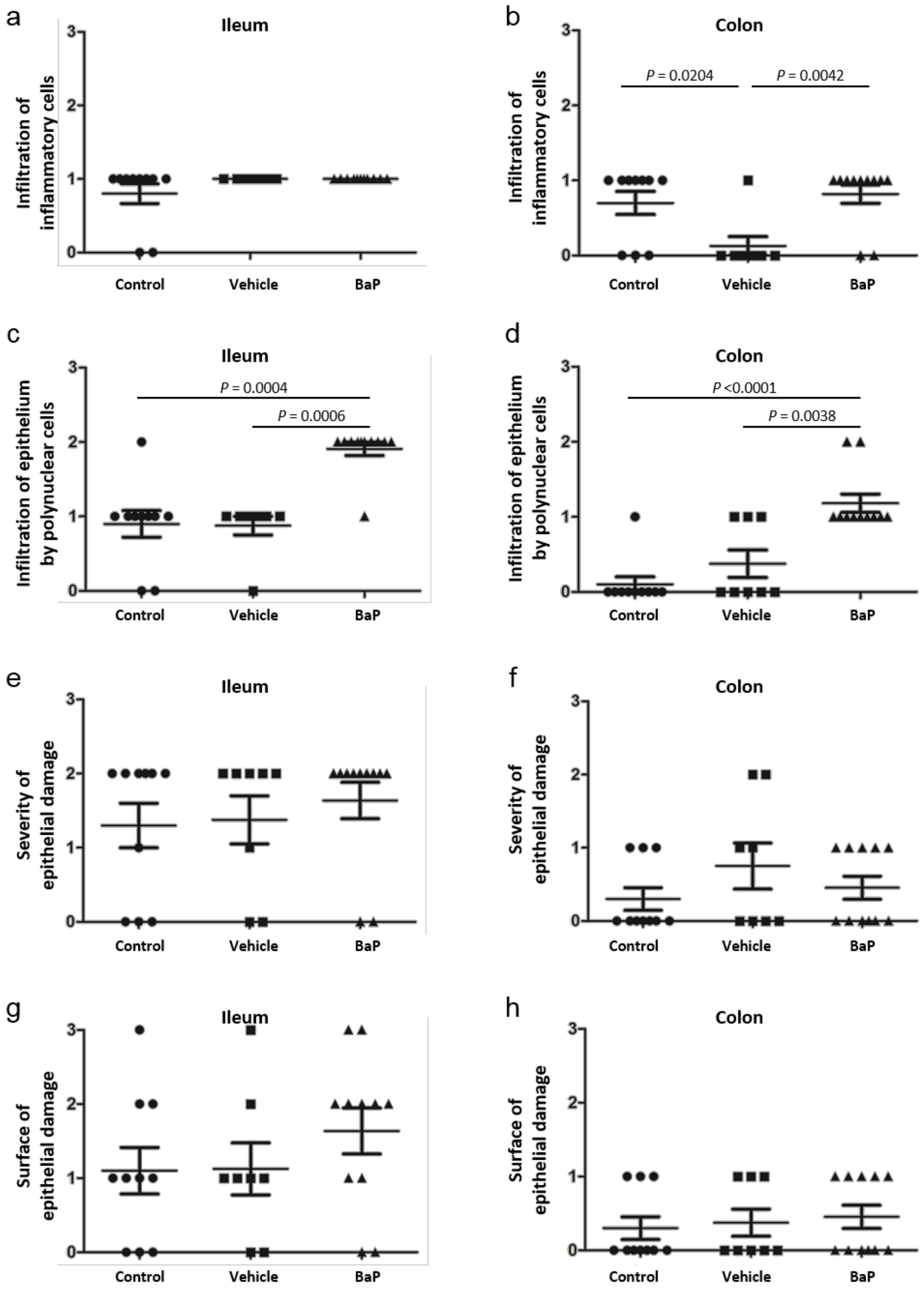

Fig S1. The score of each criterion used in the combined histological scoring of inflammation for the ileum and colon of mice. (a-b) Infiltration of inflammatory cells, (c-d) infiltration of epithelium by polynuclear cells, (e-f) severity of epithelium damage and (g-h) surface of epithelium damage of mouse ileum and colon orally exposed to sterile physiological saline (control), sunflower seed oil (vehicle) or benzo[a]pyrene (BaP). Each symbol represents an individual mouse. The values are represented with mean ± SEM and p-values are derived from a Mann-Whitney test.

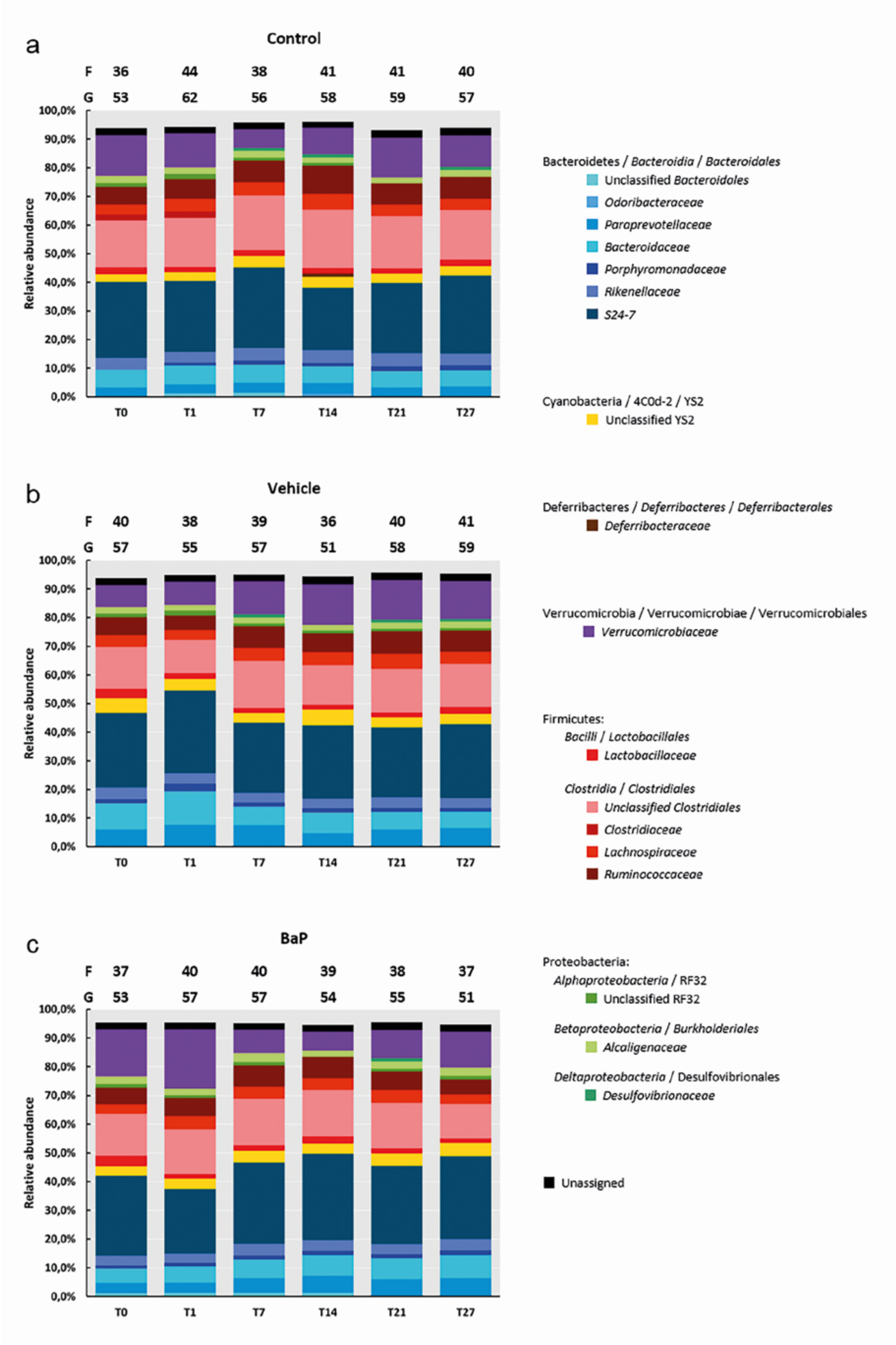

Fig S2. Faecal microbiota composition profiles at the family level in mice for control (a), vehicle (b) and BaP-treated groups (c) revealed by 16S rRNA gene sequencing. Bar plots are presented by group. Only the dominant bacterial taxa (relative abundance >1%) are represented. Families within the same bacterial phylum are indicated using different shades of the same color. The numbers above the profiles correspond to the total numbers of bacterial families (F, first line) and genera (G, second line) observed for each stool sample.

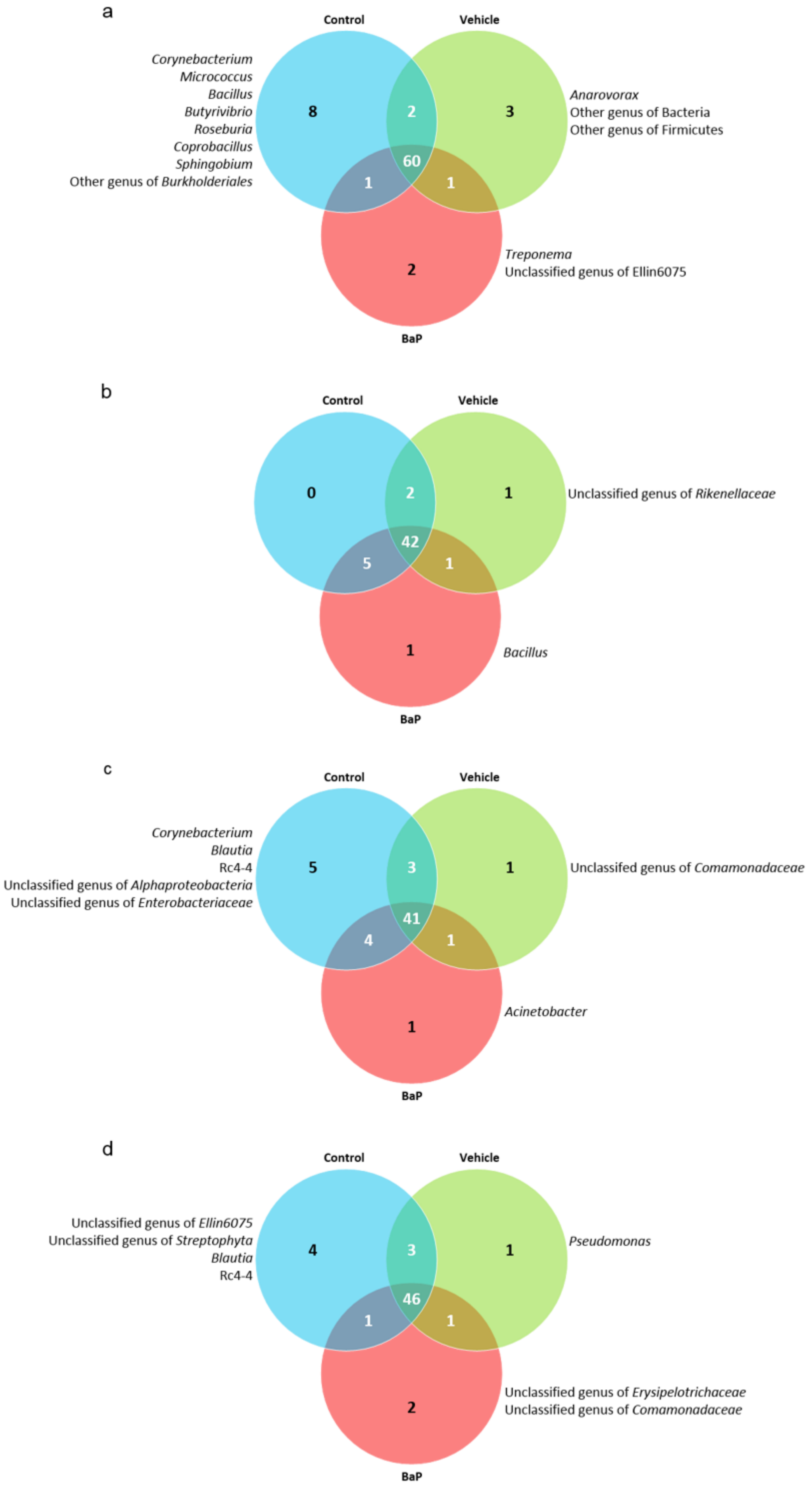

Fig S3. Shared and specific bacterial genera detected in control, vehicle and BaP-treated groups for faecal (a) and mucosa-associated microbiota in the ileum (b), proximal colon (c) and distal colon (d). Specific bacterial genera are listed next to the circle corresponding to the group in which they were detected.

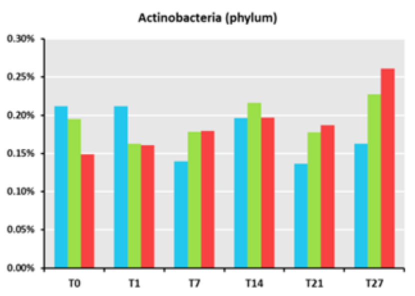
Actinobacteria (phylum)
T0 T1 T7 T14 T21 T27

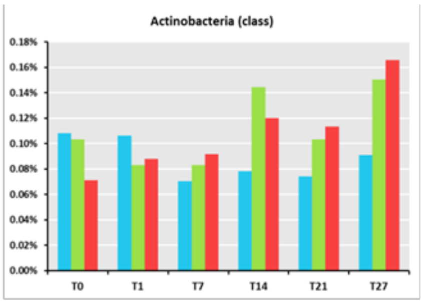
Actinobacteria (class)
T0 T1 T7 T14 T21 T27

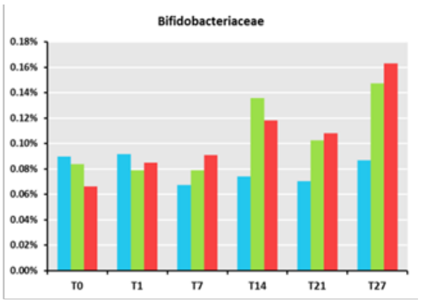
Bifidobacteriaceae
T0 T1 T7 T14 T21 T27

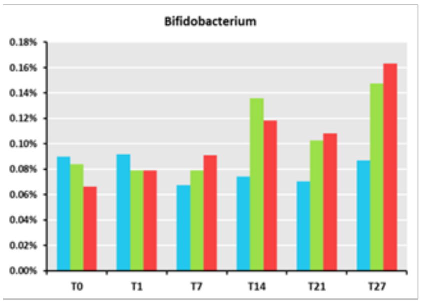
Bifidobacterium
T0 T1 T7 T14 T21 T27

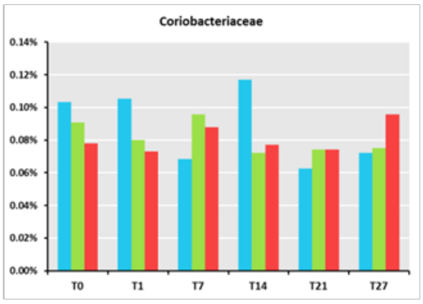
Coriobacteriaceae
T0 T1 T7 T14 T21 T27

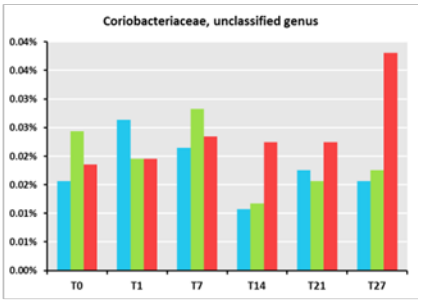
Coriobacteriaceae, unclassified genus
T0 T1 T7 T14 T21 T27

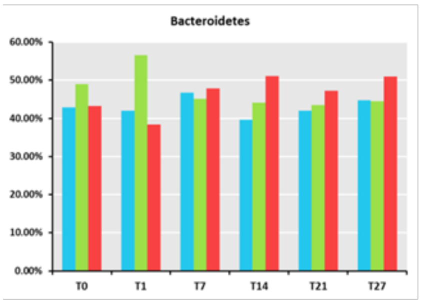
Bacteroidetes
T0 T1 T7 T14 T21 T27

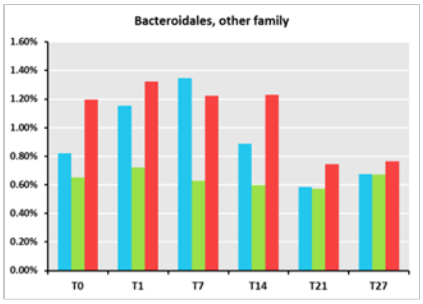
Bacteroidales, other family
T0 T1 T7 T14 T21 T27

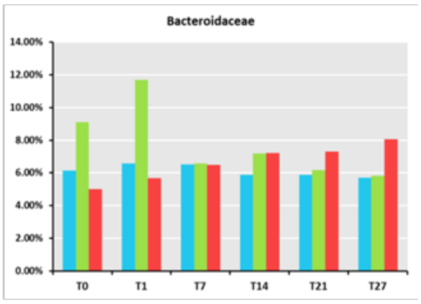
Bacteroidaceae
T0 T1 T7 T14 T21 T27

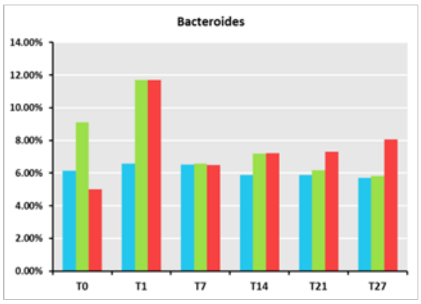
Bacteroides
T0 T1 T7 T14 T21 T27

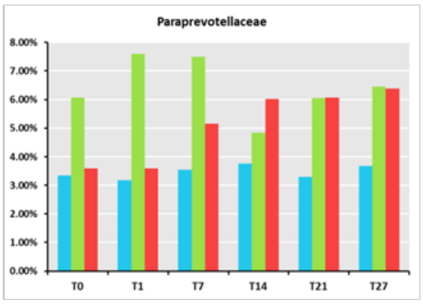
Paraprevotellaceae
T0 T1 T7 T14 T21 T27

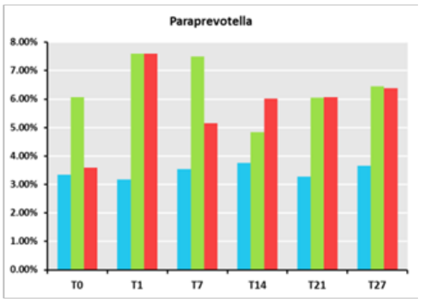
Paraprevotella
T0 T1 T7 T14 T21 T27

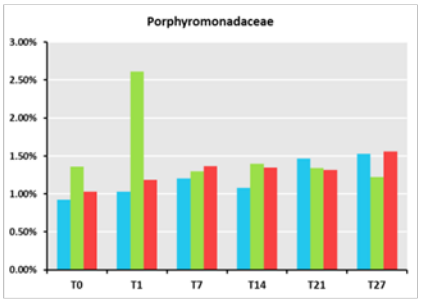
Porphyromonadaceae
T0 T1 T7 T14 T21 T27

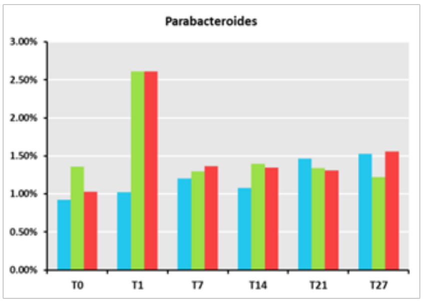
Parabacteroides
T0 T1 T7 T14 T21 T27

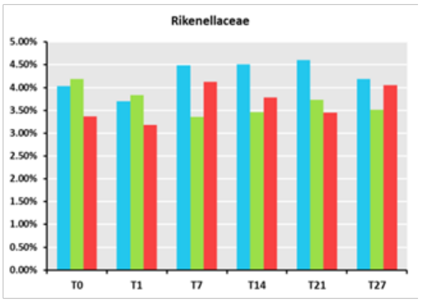
Rikenellaceae
T0 T1 T7 T14 T21 T27

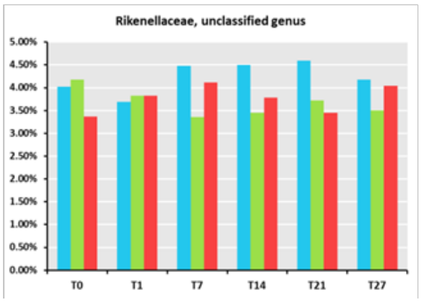
Rikenellaceae, unclassified genus
T0 T1 T7 T14 T21 T27

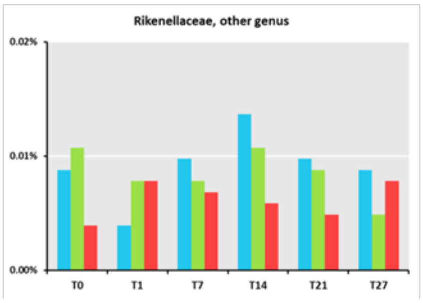
Rikenellaceae, other genus
T0 T1 T7 T14 T21 T27

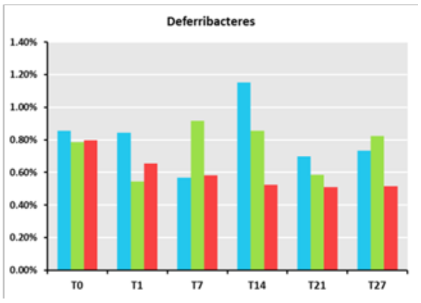
Deferribacteres
T0 T1 T7 T14 T21 T27

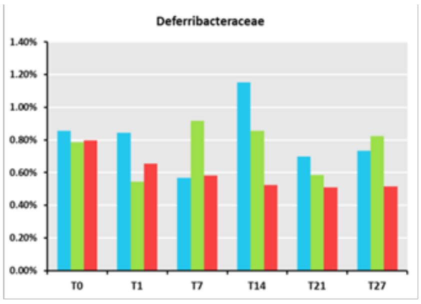
Deferribacteraceae
1.40%
1.20%
1.00%
0.80%
0.60%
0.40%
0.20%
0.00%
T0 T1 T7 T14 T21 T27

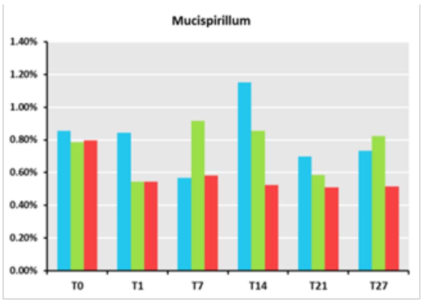
Mucispirillum
1.40%
1.20%
1.00%
0.80%
0.60%
0.40%
0.20%
0.00%
T0 T1 T7 T14 T21 T27

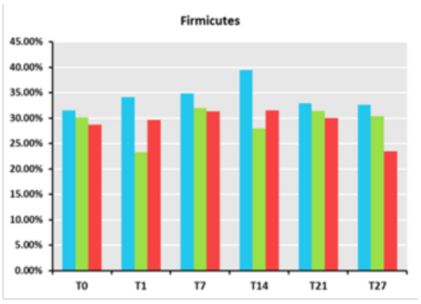
Firmicutes
45.00%
40.00%
35.00%
30.00%
25.00%
20.00%
15.00%
10.00%
5.00%
0.00%
T0 T1 T7 T14 T21 T27

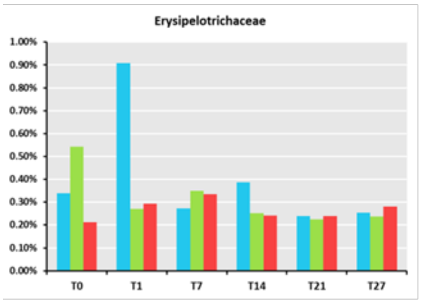
Erysipelotrichaceae
1.00%
0.90%
0.80%
0.70%
0.60%
0.50%
0.40%
0.30%
0.20%
0.10%
0.00%
T0 T1 T7 T14 T21 T27

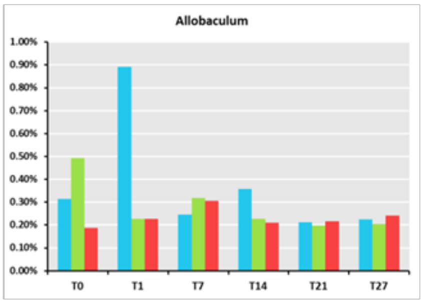
Allobaculum
1.00%
0.90%
0.80%
0.70%
0.60%
0.50%
0.40%
0.30%
0.20%
0.10%
0.00%
T0 T1 T7 T14 T21 T27

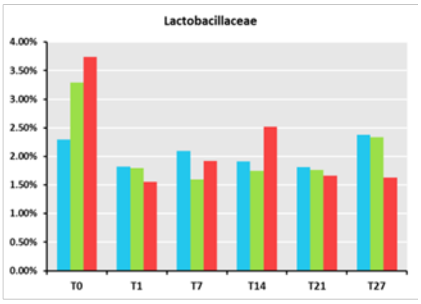
Lactobacillaceae
4.00%
3.50%
3.00%
2.50%
2.00%
1.50%
1.00%
0.50%
0.00%
T0 T1 T7 T14 T21 T27

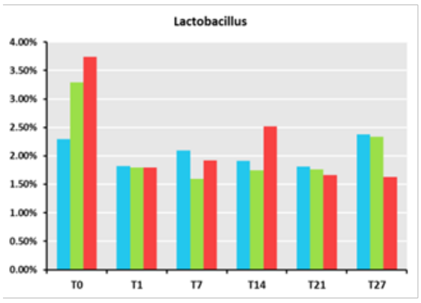
Lactobacillus
4.00%
3.5%
3.0%
2.5%
2.0%
1.5%
1.0%
0.5%
0.0%
T0 T1 T7 T14 T21 T27

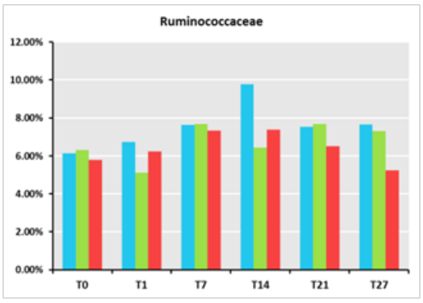
Ruminococcaceae
12.00%
10.00%
8.00%
6.00%
4.00%
2.00%
0.00%
T0 T1 T7 T14 T21 T27

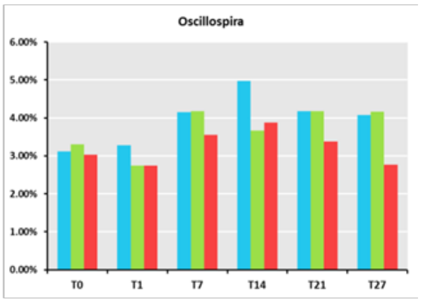
Oscillospira
6.00%
5.00%
4.00%
3.00%
2.00%
1.00%
0.00%
T0 T1 T7 T14 T21 T27

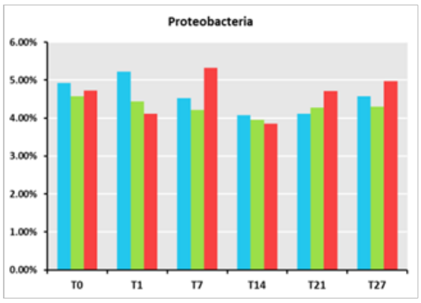
Proteobacteria
6.00%
5.00%
4.00%
3.00%
2.00%
1.00%
0.00%
T0 T1 T7 T14 T21 T27

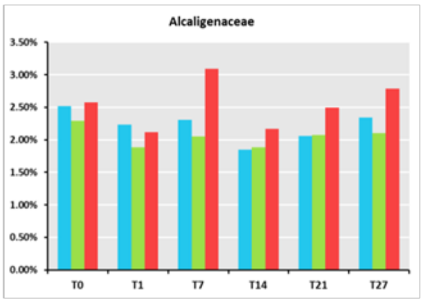
Alcaligenaceae
3.50%
3.00%
2.50%
2.00%
1.50%
1.00%
0.50%
0.00%
T0 T1 T7 T14 T21 T27

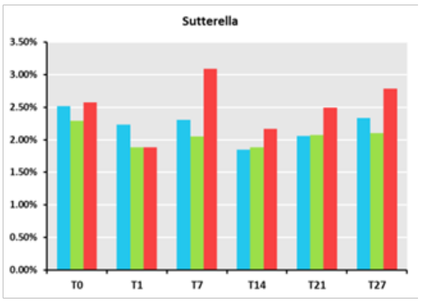
Sutterella
3.50%
3.00%
2.50%
2.00%
1.50%
1.00%
0.50%
0.00%
T0 T1 T7 T14 T21 T27

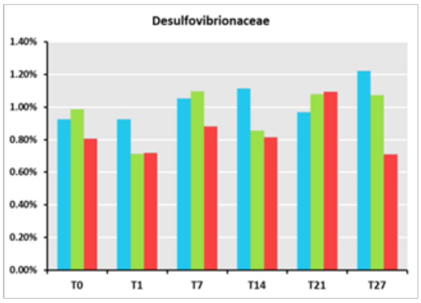
Desulfovibrionaceae
1.40%
1.20%
1.00%
0.80%
0.60%
0.40%
0.20%
0.00%
T0 T1 T7 T14 T21 T27

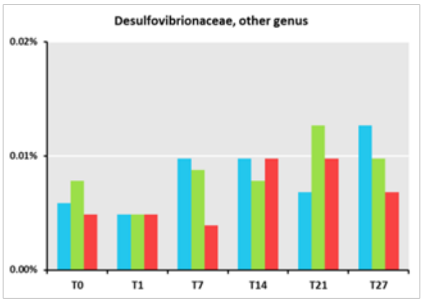
Desulfovibrionaceae, other genus
0.02%
0.01%
0.00%
T0 T1 T7 T14 T21 T27

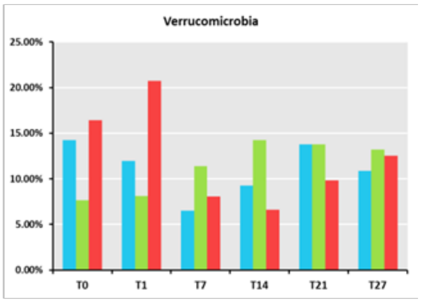
Verrucomicrobia
25.00%
20.00%
15.00%
10.00%
5.00%
0.00%
T0 T1 T7 T14 T21 T27

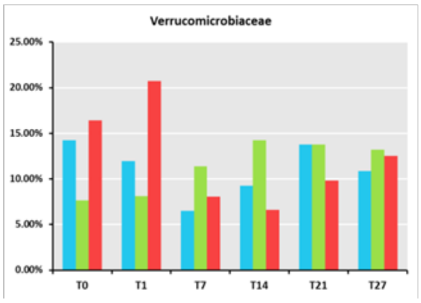
Verrucomicrobiaceae
25.00%
20.00%
15.00%
10.00%
5.00%
0.00%
T0 T1 T7 T14 T21 T27

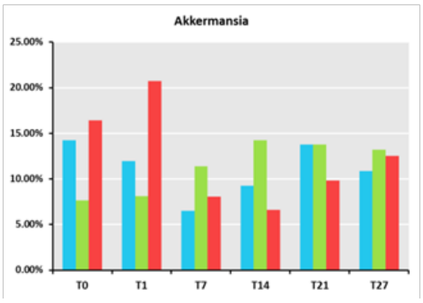
Akkermansia
25.00%
20.00%
15.00%
10.00%
5.00%
0.00%
T0 T1 T7 T14 T21 T27

Fig S4. Temporal variations (from T0 to T27) of interesting taxa relative abundance in faecal microbiota in mice for control (blue), vehicle (green) and BaP-treated groups (red) revealed by 16S rRNA gene sequencing. Bar plots are presented by taxa.

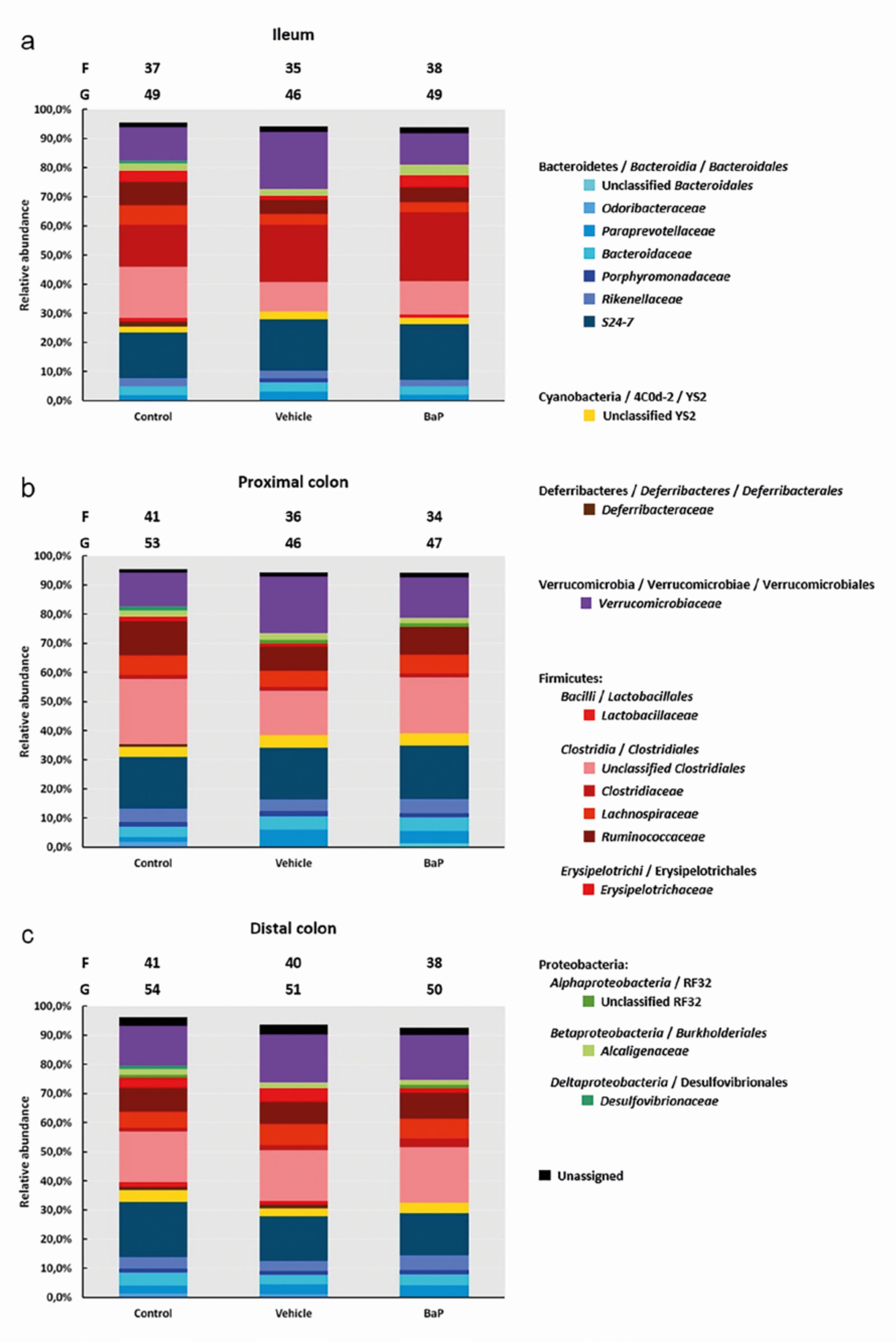

Fig S5. Mucosa-associated microbiota composition profiles at the family level in the ileum (a), proximal colon (b) and distal colon (c) in mice revealed by 16S rRNA gene sequencing. Bar plots are presented by organ localization. Only the dominant bacterial taxa (relative abundance >1%) are represented. Families within the same bacterial phylum are indicated by different shades of the same color. The numbers above the profiles correspond to the total numbers of bacterial families (F, first line) and genera (G, second line) observed for each tissue sample.

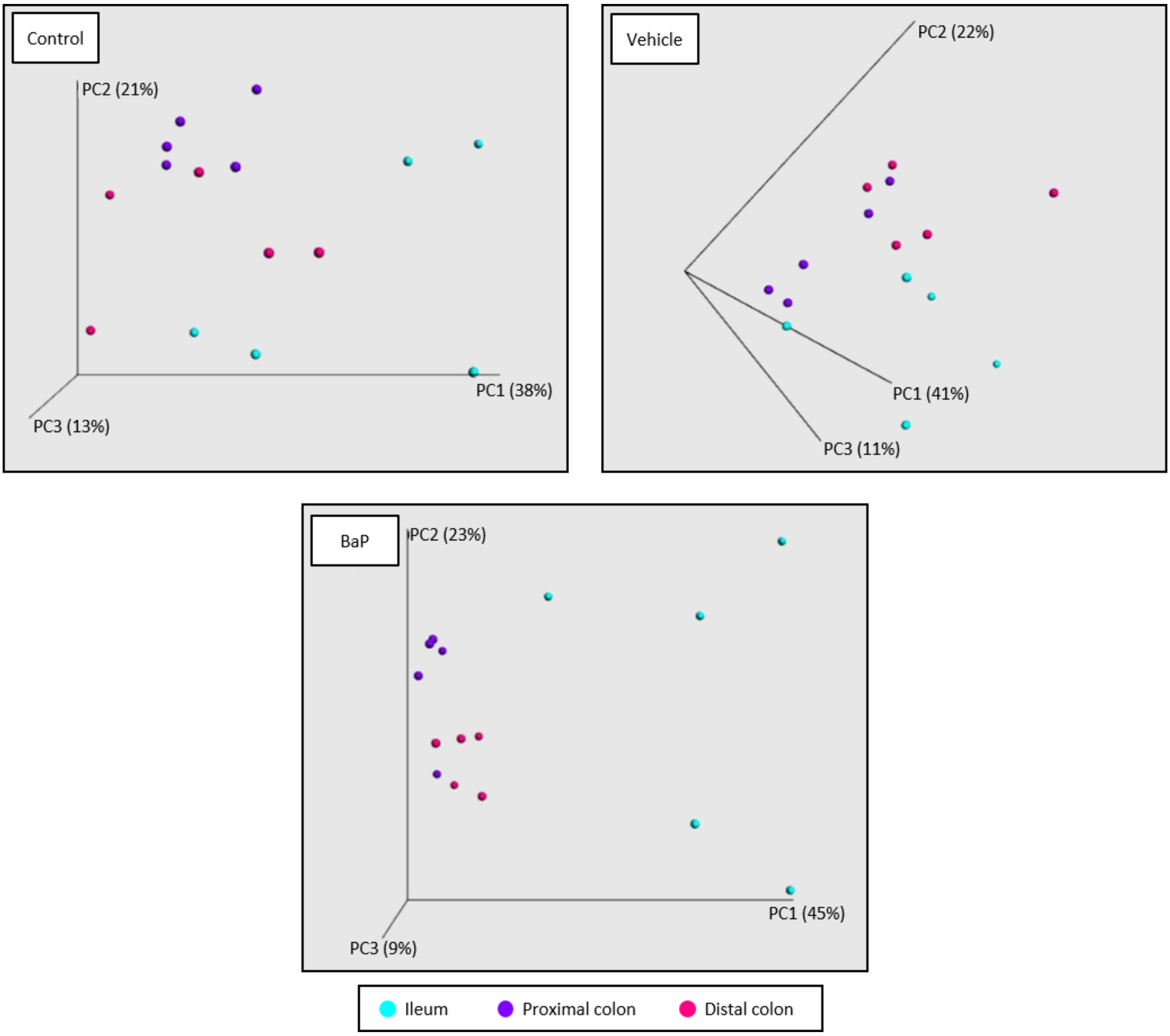

Fig S6. Mucosa-associated bacteria of the ileum, proximal colon and distal colon for control, vehicle and BaP-treated mice differentiated by principal coordinate analysis (PCoA) on a weighted UniFrac distance matrix. The ileum, proximal and distal colon were represented in blue, purple and pink respectively.
